# Supplementary material for: No Change of Pneumocystis jirovecii Pneumonia after the COVID-19 Pandemic: Multicenter Time-Series Analyses
Source: J Fungi (Basel). 2021 Nov 19;7(11):990. doi: 10.3390/jof7110990 (PMC8624436; doi:10.3390/jof7110990)
Supplement: Supplementary file 1 [file jof-07-00990-s001.zip › jof-1425950-supplementary.pdf]

Journal of Fungi

MDPI

Table S1. Non-pharmacological interventions including social distancing imposed and controlled by Korean government (Central Quarantine and Countermeasure Headquarters) during COVID-19 pandemic.

<table>
<tr><th colspan="2">Periods</th><th colspan="20">Social distancing system</th></tr>
<tr><td colspan="2" rowspan="2">January 2020
~ June 2020</td><td colspan="20">Enhanced social distancing</td></tr>
<tr><td colspan="20">● <u>Stay at home as much as possible</u>.

● <u>Call to actions for all citizens</u>
• Cancel or postpone all non-essential gathering, dining out, social events, and travel plans.
• Avoid leaving home except to purchase necessities, to get medical care, or to go to work.
• Avoid handshakes and other forms of physical contact. Keep a 2-meter distance from each other.
• Wash your hands, cover up your sneezes/coughs, and generally. Maintain strict personal hygiene.
• Disinfect and ventilate your space every day.
• If you have fever, cough, sore throat, or other respiratory symptoms, do not go to work. Stay home and get sufficient rest.

● <u>Call to actions for citizens in the workplace</u>
• Wash your hands thoroughly with soap under running water.
• Use your own personal cups and utensils.
• Refrain from using changing rooms, indoor break rooms, and other public areas.
• Avoid handshakes and other forms of physical contact. Keep a 2-meter distance from each other.
• When eating meals together, maintain a distance and avoid sitting face to face.
• Return home directly after leaving work.

● <u>It is recommended to wear a mask in the following cases</u>.
• Carrying for a COVID-19 patient
• Having respiratory symptoms such as cough, sneezing, sputum, rhinorrhea, sore throat
• Visiting medical institutions, pharmacies, seniors, disabled people, etc.
• Working in a profession that requires contact with many people
• When individuals with poor health or underlying medical conditions contact with other people within 2-meter in a poorly ventilated space
• When using indoor multi-use facilities or it is not possible to keep a distance of 2-meter outdoors</td></tr>
<tr><td colspan="2"></td><td colspan="20">Social distancing levels</td></tr>
<tr><td rowspan="7">July 2020
~ October 2020</td><td></td><td colspan="5">1</td><td colspan="5">2</td><td colspan="5">3</td><td colspan="5">4</td></tr>
<tr><td>Definition</td><td colspan="5">Maintain a state of continuous restraint</td><td colspan="5">Regional epidemic/People restrictions</td><td colspan="5">Provincial epidemic /prohibition gathering</td><td colspan="5">Nationwide pandemic/No going out</td></tr>
<tr><td>Criteria</td><td colspan="5">< 1 cases[a]/100,000</td><td colspan="5">1 ≤ cases[a]/100,000 < 2</td><td colspan="5">2 ≤ cases[a]/100,000 < 4</td><td colspan="5">≥ 4 cases[a]/100,000</td></tr>
<tr><td>Personal gathering</td><td colspan="5">Compliance with quarantine rules</td><td colspan="5">No gatherings of ≥ 9 individuals</td><td colspan="5">No gatherings of ≥ 5 individuals</td><td colspan="5">No gatherings of ≥ 3 individuals (permission of 2 individuals after 6 PM)</td></tr>
<tr><td>Events</td><td colspan="5">Prior notification to local governments for events with ≥ 500 people</td><td colspan="5">Prohibition of events with ≥ 100 people</td><td colspan="5">Prohibition of events with ≥ 50 people</td><td colspan="5">Prohibition of any events</td></tr>
<tr><td>Nightlife entertainment facilities</td><td colspan="5">No limitation</td><td colspan="5">Restriction of operation after midnight</td><td colspan="5">Restriction of operation after 10 PM</td><td colspan="5">Prohibition of gathering</td></tr>
<tr><td>Restaurants and cafes</td><td colspan="5">No limitation</td><td colspan="5">Permission of only packaging and delivery after midnight</td><td colspan="5">Permission of only packaging and delivery after 10 PM</td><td colspan="5">Permission of only packaging and delivery after 10 PM</td></tr>
<tr><td rowspan="7">November 2020
~ June 2021</td><td></td><td colspan="4">1</td><td colspan="4">1.5</td><td colspan="4">2</td><td colspan="4">2.5</td><td colspan="4">3</td></tr>
<tr><td rowspan="3">Definition and major call to actions</td><td colspan="4">Life quarantine</td><td colspan="8">Local epidemic stage</td><td colspan="8">National epidemic stage</td></tr>
<tr><td colspan="4">< 100 cases[a] in Seoul Metropolitan area</td><td colspan="4">≥ 100 cases[a] in Seoul Metropolitan area</td><td colspan="4">≥ doubled over 1.5 stage or ≥ 300 cases[a] nationwide</td><td colspan="4">≥ 400~500 cases[a] nationwide or rapid increase in patients</td><td colspan="4">≥ 800~1000 cases[a] nationwide or rapid increase in patients</td></tr>
<tr><td colspan="4">● Compliance with quarantine rules for COVID-19 prevention while maintain daily life and socioeconomic activity</td><td colspan="4">● Start of local epidemic
● Through quarantine in dangerous areas</td><td colspan="4">● Rapid propagation of local epidemic
● Restraint of unnecessary going out and gatherings in the risk area</td><td colspan="4">● Spread nationwide epidemic
● Preferably stay at home and refrain from going out, meetings and using multi-use facilities as much as possible</td><td colspan="4">● Nationwide pandemic
● As a rule, stay at home and minimize contact with others</td></tr>
<tr><td>Nightlife entertainment facilities</td><td colspan="4">Limited to 1 person per 4 m^2</td><td colspan="4">Adding no movement between seats</td><td colspan="12">Prohibition of gathering</td></tr>
<tr><td>Restaurants and cafes</td><td colspan="4">1 meter distance between tables in places over 150 m^2</td><td colspan="4">Expanding the distance in places over 50 m^2</td><td colspan="8">Adding only permission of packaging and delivery after 9 PM</td><td colspan="4">Adding the limitation to 1 person per 8 m^2</td></tr>
<tr><td>Mandatory to wear a mask (Penalties for violations)</td><td colspan="4">All indoor facilities including public transportation</td><td colspan="4">Adding outdoor sports arena</td><td colspan="4">All indoor + outdoor with high-risk activities</td><td colspan="8">All indoor + outdoors where it is difficult to keep a distance of 2 meters</td></tr>
</table>

**Meeting and events**	Consultation with local governments for gatherings of ≥ 500 + Mandatory observance of basic quarantine rules	No gatherings of ≥ 100 people at special events such as festivals	No gatherings of ≥ 100 people	No gatherings of ≥ 50 people	No gatherings of ≥ 10 people
**Working at workplace**	Recommendation to activate telecommuting/homeworking			≥ 2/3 recommended to work from home	Mandatory telecommuting except for essential personnel
**Going to school**	1/3 of the density, in principle	Observe 2/3 of the density	1/3 of the density, in principle	Observe 1/3 of the density	Full remote learning

[a]Average PCR-confirmed patients per week.

References:

1. Korea Centers for Disease Control and Prevention. Enhanced social distancing campaign. 2020.4.4. http://ncov.mohw.go.kr/searchBoardView.do?brdId=3&brdGubun=32&dataGubun=321&ncvContSeq=1497
2. Central Quarantine and Countermeasure Headquarters. The COVID-19 Guidelines of the Republic of Korea for distancing in daily life. http://ncov.mohw.go.kr/en/guidelineView.do?brdId=18&brdGubun=181&dataGubun=&ncvContSeq=2763&contSeq=2763&board_id=&gubun=#
3. Central Quarantine and Countermeasure Headquarters. Overview of Social Distancing System. http://ncov.mohw.go.kr/en/socdiBoardView.do?brdId=19&brdGubun=191&dataGubun=191&ncvContSeq=&contSeq=&board_id=&gubun=

Table S2. Numbers of PCP-confirmed and suspected inpatients, and PCP rates by month and year.

	2015			2016			2017			2018			2019			2020			2021			Total[a]		
	**S**	**C**	**R**	**S**	**C**	**R**	**S**	**C**	**R**	**S**	**C**	**R**	**S**	**C**	**R**	**S**	**C**	**R**	**S**	**C**	**R**	**S[a]**	**C[a]**	**R[a]**
**January**	199	29	**14.6**	181	30	**16.6**	202	27	**13.4**	323	50	**15.5**	308	33	**10.7**	317	47	**12.7**	337	40	**11.9**	274.4	36.6	**13.6**
**February**	187	18	**9.6**	177	32	**18.1**	214	37	**17.3**	220	34	**15.5**	277	29	**10.5**	275	38	**13.8**	317	40	**12.6**	238.1	32.6	**13.9**
**March**	251	29	**11.6**	166	22	**13.3**	226	32	**14.2**	260	42	**16.2**	270	36	**13.3**	365	50	**13.7**	353	49	**13.9**	270.1	37.1	**13.7**
**April**	227	31	**13.7**	162	33	**20.4**	230	33	**14.3**	264	32	**12.1**	311	36	**11.6**	310	37	**11.9**	344	47	**13.7**	264.0	35.6	**14.0**
**May**	185	33	**17.8**	175	41	**23.4**	269	30	**11.2**	298	49	**16.4**	303	52	**17.2**	312	41	**13.1**	337	55	**16.3**	268.4	43.0	**16.5**
**June**	132	25	**18.9**	214	40	**18.7**	216	29	**13.4**	310	43	**13.9**	312	68	**21.8**	311	49	**15.8**	330	53	**16.1**	260.7	43.9	**16.9**
**July**	166	28	**16.9**	205	26	**12.7**	234	23	**9.8**	277	57	**20.6**	308	59	**19.2**	326	45	**13.8**	—	—	—	252.7	39.7	**15.5**
**August**	133	23	**17.3**	181	26	**14.4**	255	25	**9.8**	288	36	**12.5**	320	54	**16.9**	256	34	**13.3**	—	—	—	238.8	33.0	**14.0**
**September**	141	29	**20.6**	157	29	**18.5**	265	33	**12.5**	309	49	**15.9**	290	48	**16.6**	296	40	**13.5**	—	—	—	243.0	38.0	**16.2**
**October**	168	29	**17.3**	169	36	**21.3**	208	20	**9.6**	278	47	**16.9**	316	53	**16.8**	309	26	**8.4**	—	—	—	241.3	35.2	**15.0**
**November**	152	29	**19.1**	183	38	**20.8**	207	29	**14.0**	282	43	**15.2**	316	53	**16.8**	339	30	**8.8**	—	—	—	246.5	37.0	**15.8**
**December**	171	35	**20.5**	265	43	**16.2**	263	36	**13.7**	335	28	**8.4**	281	44	**15.7**	393	38	**9.7**	—	—	—	284.7	37.3	**14.0**
**Total[a]**	176	28	**16.5**	186	33	**17.9**	232	30	**12.8**	287	43	**14.9**	301	47	**15.6**	322	40	**12.4**	336	47	**14.1**	**256.9**	**37.4**	**14.9**

S, C, and R indicate the PCP-suspected and confirmed inpatients, and PCP rates, respectively. [a]Average value.

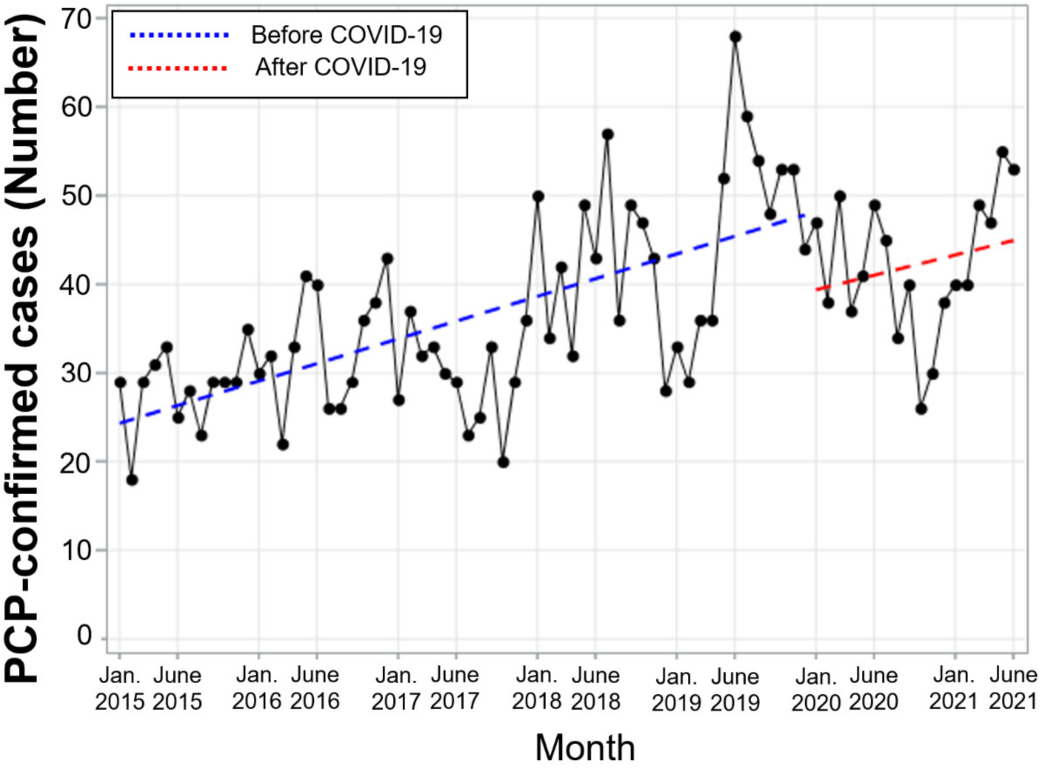

Figure S1. Plots and liner regression lines of the monthly observed PCP cases in all PCP-confirmed inpatients in the pre- and post-COVID-19 periods. The dots and dotted lines indicate the observed PCR cases and linear regression lines, respectively.

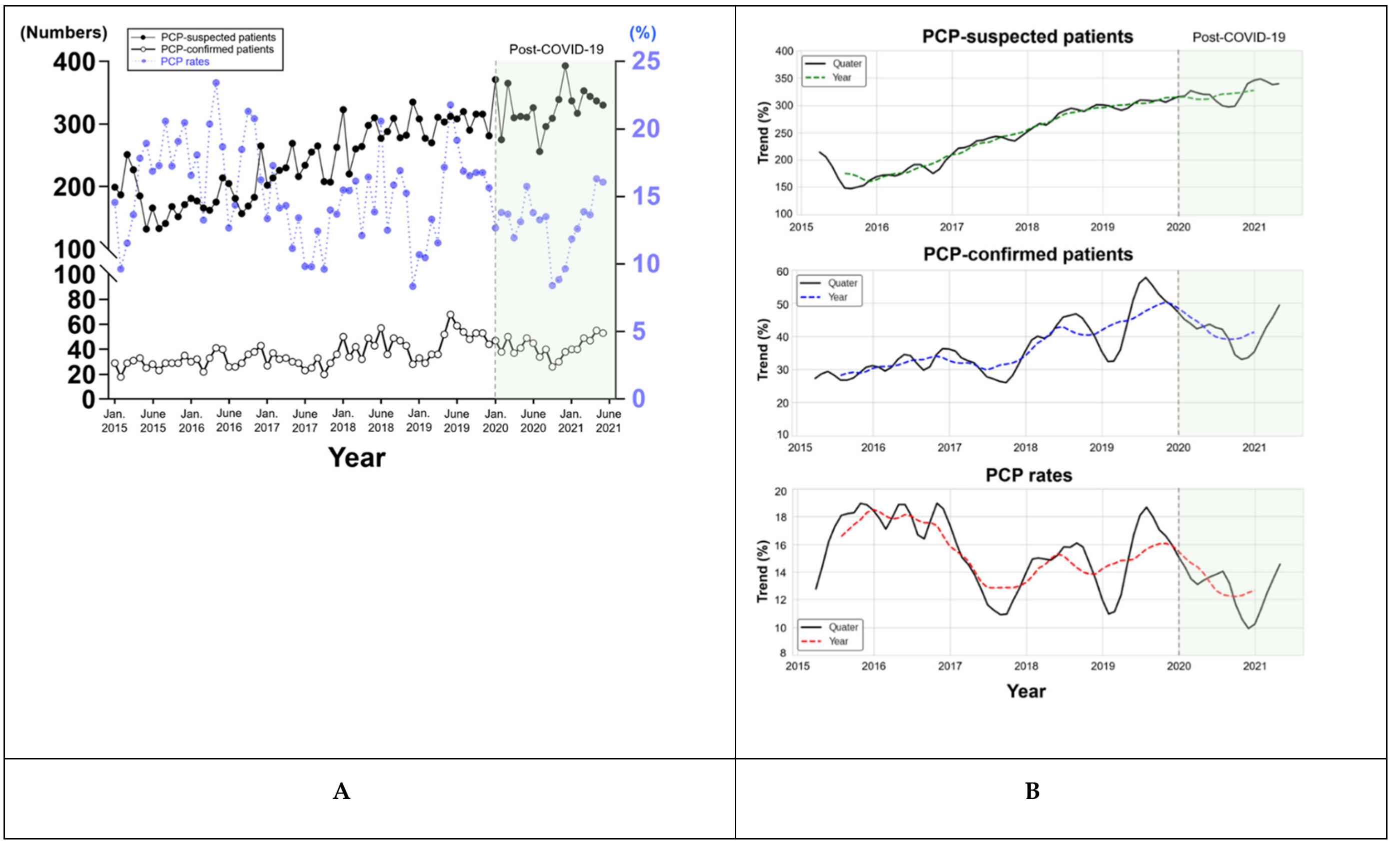

A	B

Figure S2. The observed data and trends of number of PCP-suspected and confirmed inpatients, and PCP rates by seasonal decomposition method of the ETS model. The number of PCP-suspected or-confirmed inpatients and PCP rates are plotted

on the left and right Y-axis, respectively, in (A). Abbreviations: PCP, *P. jirovecii* pneumonia. (**A**) Observed data and (**B**) Trends.

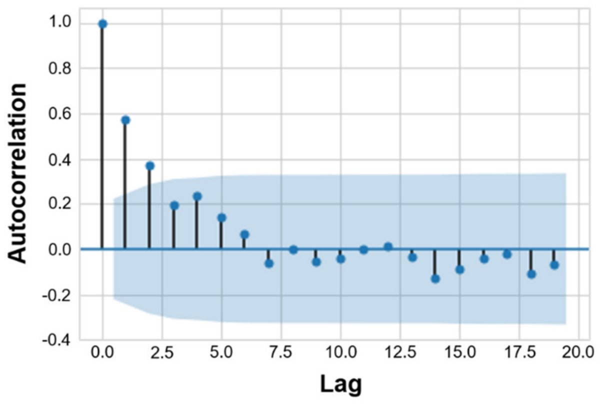	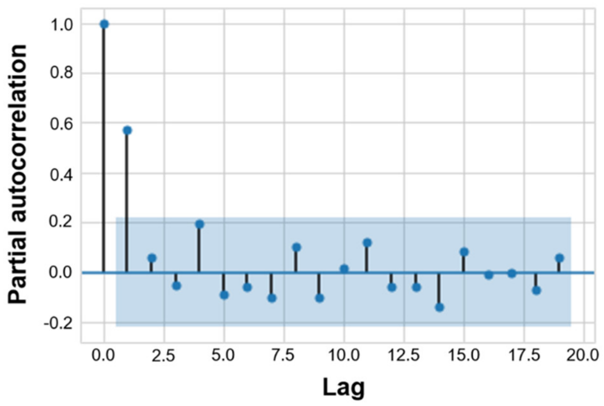
**A**	**B**

Figure S3. Autocorrelation and partial autocorrelation functions of residuals in the ARIMA model. (**A**) Autocorrelation and (**B**) Partial autocorrelation.The plots of autocorrelation and partial autocorrelation were obtained from the statsmodels package in Python. Aberrations: ARIMA, autoregressive integrated moving average.
